# Illuminating the Effect of the Local Environment on the Performance of Organic Sunscreens: Insights From Laser Spectroscopy of Isolated Molecules and Complexes

**DOI:** 10.3389/fchem.2021.812098

**Published:** 2022-01-12

**Authors:** Natalie G. K. Wong, Caroline E. H. Dessent

**Affiliations:** Department of Chemistry, University of York, York, United Kingdom

**Keywords:** sunscreens, lasers, pH, solvent, rational design, photochemistry, photophysics

## Abstract

Sunscreens are essential for protecting the skin from UV radiation, but significant questions remain about the fundamental molecular-level processes by which they operate. In this mini review, we provide an overview of recent advanced laser spectroscopic studies that have probed how the local, chemical environment of an organic sunscreen affects its performance. We highlight experiments where UV laser spectroscopy has been performed on isolated gas-phase sunscreen molecules and complexes. These experiments reveal how pH, alkali metal cation binding, and solvation perturb the geometric and hence electronic structures of sunscreen molecules, and hence their non-radiative decay pathways. A better understanding of how these interactions impact on the performance of individual sunscreens will inform the rational design of future sunscreens and their optimum formulations.

## Introduction

The incidence of melanoma skin cancer has reached epidemic proportions globally, with cases predicted to continue rising by 2.5% year on year. Against this background, considerable effort is being made towards developing more efficient broad-spectrum sunscreens that protect against both UVA and high-energy UVB rays (Forestier, 2008; Campos et al., 2017). Despite the importance of sunscreens to human health remarkably little was known until very recently about how photoactive organic sunscreens function in terms of their detailed molecular potential energy surfaces (Karsili et al., 2014; Baker et al., 2017). An organic sunscreen molecule works by absorbing damaging UV radiation, and dispersing it into less harmful forms of energy (Forestier, 2008; Karsili et al., 2014; Baker et al., 2017; Losantos et al., 2018). One key requisite for such molecules to act as chemical sunscreens is that they should not undergo chemical change or induce unwanted toxicity upon exposure to UV light (Forestier, 2008). However, the extent to which a molecule’s structure remains intact after absorbing UV light depends not only on its intrinsic photochemistry, but also on how that photochemistry is affected by external chemical and physical influences such as pH, aggregation between components in a mixture, and the effects of phase change.

Commercial sunscreen lotions are complex multicomponent mixtures of organic and inorganic substances dispersed in a mixture of solvents (Osterwalder et al., 2014). This complexity means that individual organic sunscreen molecules can experience a range of different intermolecular interactions within the suncream formulation, including interactions with solvent, counterions and other organic sunscreen molecules. All of these interactions have the potential to perturb the electronic structure of the organic sunscreen, and hence its intrinsic photochemistry. Sunscreen development in commercial laboratories tends to focus on achieving an acceptable bulk formulation without considering such molecular-level interactions (Acker et al., 2014). However, it is clear that a better fundamental understanding of how intermolecular interactions can impact on the performance of organic sunscreens could be beneficial in the rational design of new and improved sunscreens and their optimum formulations (Serpone, 2021). Over recent years, laser spectroscopy techniques have been applied to better understand how intermolecular interactions (e.g., with solvent molecules) and the local environment (pH) affect sunscreen photochemistry. These studies have either been performed in highly simplified mixtures (i.e., one sunscreen molecule and one solvent) or on individual molecules and their complexes in the gas phase (Tan et al., 2014; Rodrigues and Stavros, 2018). In this review, we provide an overview of recent work in this area and illustrate how such studies are beginning to impact on the development of new sunscreen agents.

Before moving to discuss the impact of the molecular-level environment on sunscreen photochemistry, it is useful to review the molecular properties that are linked to good sunscreen action. The majority of organic sunscreen molecules are composed of structures that contain aromatic rings conjugated to carbonyl groups, with examples including cinnamates, salicylates, oxybenzone and avobenzone (Forestier, 2008). All of these molecules provide photoprotection through a combination of a high absorption cross-section for UV light coupled with high internal conversion (IC) efficiency (Forestier, 2008; Baker et al., 2017; Rodrigues and Stavros, 2018). UV electronic excitation is therefore followed by rapid conversion to vibrational energy which is dissipated as heat to the molecule’s surroundings. The extent to which the UV energy absorbed is converted to benign heat is a measure of the suitability of the molecule as a sunscreen, since it ensures that the molecule is able to absorb and dissipate repeated UV photons. Ideally, a sunscreen molecule should be able to dissipate excited state energy through IC (i.e., non-radiatively) on a rapid timescale (femtoseconds-picoseconds), to reduce the possibility of harmful side reactions such as the formation of triplet states, and/or molecular fragmentation (Serpone et al., 2007).

Common organic sunscreen molecules display a range of IC pathways that allow them to dissipate absorbed UV energy. For cinnamates, their side chains contain C=C double bonds that photoisomerize following UV absorption, thus opening an excited state pathway towards a conical intersection which facilitates IC (Peperstraete et al., 2016). Oxybenzone illustrates a different type of IC pathway: It contains an H atom donor on a hydroxyl group adjacent to a carbonyl oxygen acceptor group, allowing intramolecular hydrogen transfer. Following UV photoexcitation, excited state hydrogen transfer, which drives enol-keto tautomerisation, and leads to a slower rotation about the C-C bond which in turn facilitates IC back to the electronic ground state through an S_1_/S_0_ conical intersection (Baker et al., 2015). High-level computational studies of the ground and excited state potential energy surfaces of these sunscreen molecules confirm the pathways outlined here, with the oxybenzone system having been studied by Domcke and co-workers (Karsili et al., 2014), and the cinnamates by Cui and co-workers and Ebata and co-workers (Chang et al., 2015; Kinoshita et al., 2021). Any geometric change of the key functional groups involved in accessing the conical intersection for IC has the potential to perturb the optimum decay dynamics of the sunscreen molecule. Thus, our focus in this review will be to explore at molecular-level detail, whether local environmental effects (i.e., solvent molecules, counterions) can lead to geometric structural modifications at the key molecular functional groups, and hence perturb the molecule’s electronic structure to impact its photophysical behaviour.

## The Effect of pH on Organic Sunscreens

Despite the significant growth in fundamental studies of sunscreens over the last decade, surprisingly little attention appears to have been paid to the effect of the pH environment on sunscreen performance (De Laurentiis et al., 2013; Ignasiak et al., 2015; Li et al., 2016). From a chemical perspective, the question to be addressed is straightforward, namely how do the properties of the protonated or deprotonated sunscreen molecule differ from those of the neutral? Li et al. (2016) recently performed a series of oxybenzone photolysis studies in pure water, which revealed that while the neutral form of oxybenzone is stable over long timescales, the anionic form is not. This is potentially a key issue since commercial sunscreens typically involve complex mixtures including water and alcoholic solvents. Moreover, in common usage, sunscreens are exposed to acidic and alkaline environments, with chlorinated swimming pools and the ocean being alkaline (Kulthanan et al., 2013), while human skin and sweat are typically mildly acidic (Proksch, 2018).

Laser-interfaced mass spectrometry (LIMS) is an excellent experimental method for exploring the spectroscopy and photochemistry of protonated and deprotonated forms of the same molecular system (Matthews and Dessent, 2018; Matthews et al., 2018; Uleanya et al., 2020). Since experiments are conducted on isolated, mass-selected ions, the charged system under investigation is unambiguous, and any complications of the bulk environment are removed. The technique allows the measurement of the gas-phase absorption profile of the charged molecule, along with the photon-energy dependent production profile of any ionic photoproducts (Matthews et al., 2016). We have applied this approach over recent years to a number of protonated and deprotonated forms of organic sunscreens (Matthews and Dessent, 2017; Wong et al., 2019b; Wong et al., 2019a; Wong et al., 2021a; Wong et al., 2021b; Berenbeim et al., 2020b).

**FIGURE 1:**
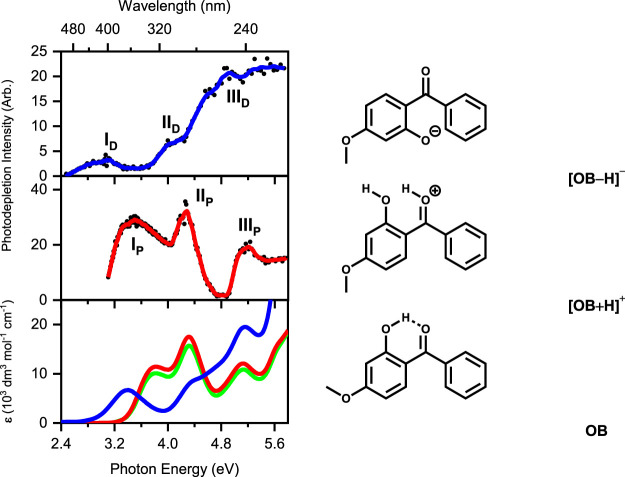
(Top) Gas-phase photodepletion (absorption) spectrum of deprotonated oxybenzone ([OB−H]^−^). (Middle) Gas-phase photodepletion spectrum of protonated oxybenzone ([OB + H]^+^). The solid lines are a 5-point adjacent average of the data points. A schematic of the lowest-energy isomers of [OB−H]^−^ and [OB + H]^+^ are shown alongside their respective spectra. (Bottom) Solution-phase absorption spectra of OB under alkaline (blue), neutral (green), and acidic (red) conditions. Data reproduced from (Wong et al., 2019b) with permission from the PCCP owner societies.

LIMS was first applied in 2019 to the protonated and deprotonated forms of an organic UV filter, oxybenzone (Wong et al., 2019b). Figure 1 displays the gas-phase absorption spectra of the protonated and deprotonated forms of oxybenzone, along with the solution-phase UV-VIS spectra. From the spectra displayed in Figure 1, it is clear that the protonation state has a dramatic effect on the absorption properties. While the UV absorption profile (400–216 nm) of oxybenzone was only modestly affected by protonation, deprotonated oxybenzone displays a considerably modified absorption spectrum, with very low absorption in the UVA region between 370–330 nm.

The photodissociation pathways of oxybenzone are also found to be affected by the protonation state, with the protonated form photofragmenting primarily by rupture of the bonds on either side of the central carbonyl group, with a significant number of additional photofragments also observed. Intriguingly, the production spectra of these various photofragments fell into two distinctive groups, in terms of their excitation energy production profiles. This revealed that two distinctive decay pathways are accessible to protonated oxybenzone across this region, possibly due to coupling of the initially accessed π-π* transition with an energetically similar charge-transfer state (Dean et al., 2014). In addition, analysis of the photofragments provided information on the nature of the excited-state decay dynamics, revealing that one of these pathways was not associated with ultrafast IC decay. This result is important as it indicates that the protonated form of oxybenzone is a non-ideal sunscreen over the UVA region, and would be likely to undergo enhanced photodegradation under acidic conditions.

Deprotonated oxybenzone photofragments in a completely different way to the protonated form, photodissociating with either loss of a methyl free radical, or a methane molecule from the starting molecule. These results for the isolated deprotonated oxybenzone ion are consistent with the results of Li et al. (2016) who observed oxybenzone acting as a photosensitiser under alkaline conditions, since the free radical photoproducts we observe in the gas-phase system could initiate the observed photosensitiser behaviour in solution.

The potential energy surfaces on which neutral oxybenzone relaxes back to the ground state after UV absorption are well-characterised following advanced quantum-chemical calculations. Domcke and co-workers found that excited-state decay involves proton transfer from the enol to keto forms, followed by rapid internal conversion (Karsili et al., 2014). The expected geometric forms of protonated and deprotonated oxybenzone predict that the keto-enol site is the protonation/deprotonation location, so it is entirely unsurprising to find that the ultrafast decay mechanism is significantly perturbed in alkaline or acidic media. Indeed, this is the key finding from the series of deprotonated and protonated sunscreen molecules we have studied *via* LIMS, which include avobenzone, 2-phenylbenzimidazole-5-sulfonic acid, and benzophenone-4 (Wong et al., 2019a; Berenbeim et al., 2020b; Wong et al., 2021a). Protonation/deprotonation will impact on the ultrafast decay mechanisms if a geometric change occurs at a structural location involved in the pathway by which the excited state accesses the conical intersection. This occurs upon protonation of avobenzone (Berenbeim et al., 2020b), but not upon deprotonation of benzophenone-4 (Wong et al., 2021a), illustrating the importance of considering the pK_a_/pK_b_’s of the sunscreen functional groups, alongside an assessment of these functional group’s involvement in the ultrafast photodecay pathway.

Finally, we note that a growing number of deprotonated/protonated sunscreen molecules have recently been studied in the gas-phase. Table 1 provides an overview of these recent studies, highlighting the sunscreens studied and the techniques used.

**TABLE 1:** Summary of Isolated (Gas-Phase) Sunscreen Molecules Studied in their Protonated or Deprotonated Forms.

Sunscreen	Protonated (P) Or Deprotonated (D)	Technique	Reference
Oxybenzone	P and D	LIMS^a^	Wong et al. (2019b)
Avobenzone	P	LIMS^a^	Berenbeim et al. (2020b)
2-phenylbenzimidazole-5-sulfonic acid	D	LIMS^a^	Wong et al. (2019a)
benzophenone-4	D	LIMS^a^	Wong et al. (2021a)
*para*-aminobenzoic acid (PABA)	P	LIMS^a^	Matthews and Dessent, 2017
*trans*-*para*-coumaric acid	D	LIMS^a^	Wong et al. (2021b)
*trans*-caffeic acid	D	LIMS^a^	Wong et al. (2021b)
*trans*-ferulic acid	D	LIMS^a^	Wong et al. (2021b)
*para*-coumaric acid	D	TRPES^b^	Bull et al. (2020)
*para*-coumaric acid	D	TRPES^b^	Henley et al. (2018)
*para*-coumaric acid	D	LP-IMMS^c^	Bull et al. (2019)

a LIMS: Laser Interfaced Mass Spectrometry.

b TRPES: Time Resolved Photoelectron Spectroscopy.

c LP-IMMS: Laser Photodissociation Ion Mobility Mass Spectrometry.

## Sodium Cation Binding Can Disrupt Sunscreen Action

Alkali metal cations are common constituents of commercial sunscreen mixtures, where they are typically coupled to pH buffered anions. They are also commonly encountered in high concentrations in environments were sunscreens are used, such as swimming pools and the sea. Given that protonation can impact on the function of an organic sunscreen at the molecular level, it is clearly important to understand if cation binding could produce a similar effect. To investigate this, a series of experiments were performed on isolated complexes, M^+^·OB, where alkali metal cations (M^+^ = Na^+^, K^+^ and Rb^+^) were bound to the sunscreen oxybenzone, OB (Berenbeim et al., 2020a).

The electronic spectrum of the Na^+^·OB complex was found to be strikingly different from those of K^+^·OB and Rb^+^·OB, indicating that the Na^+^ cation binds to OB with a different binding motif than K^+^ and Rb^+^. Infrared multiphoton dissociation (IRMPD) spectroscopy conducted at the FELIX free electron facility and computational calculations revealed that the cation-dependent UV spectra could be traced to the compact Na^+^ ion breaking the intramolecular hydrogen bond in OB, and adopting the position normally taking by the hydrogen atom in the most stable form of OB. In contrast, K^+^ and Rb^+^ appear to prefer to bind above the aromatic rings, leaving the intramolecular hydrogen bond intact. This is an important result in terms of the UV filtering ability of OB, since the disruption of the intramolecular hydrogen bond that occurs upon Na^+^ binding blocks the non-radiative relaxation mechanism that relies on excited state electron driven hydrogen atom transfer. A number of other common organic sunscreens (e.g., dioxybenzone and octyl salicylate) relay on similar intramolecular hydrogen bonding for non-radiative relaxation. The results found for the Na^+^·OB complex suggest that close coordination to Na^+^ may jeopardize the photostability of these molecules, both in sunscreen mixtures with significant sodium ion concentrations, but also in locations such as swimming pools and the sea.

In considering the potential impact of sodium ion cationization on sunscreen molecules, it is important to acknowledge that the organic sunscreen molecules are frequently present in an oil phase of the sunscreen formulation, which is suspended in an aqueous phase. As such, the sunscreens are in limited contact with metal cations which can be expected to be largely contained in the aqueous phase in the pure formulations. The extent to which phase mixing allows direct contact of cations and sunscreens will be dynamic, as well as location/climate dependent. Nonetheless, a possible enhanced degradation pathway is suggested by the results described above that merits future investigation.

To our knowledge, our study of the effect of alkali metal complexation on oxybenzone is the first to directly probe how cation binding can affect the photodynamics of a sunscreen molecule. However, our results were mirrored by other subsequent studies where metal cation binding has been reported to significantly perturb the excited-state behaviour of an aromatic molecule (Marlton et al., 2021; Robertson et al., 2021), indicating that cation coordination may have widespread photochemical importance.

## How Does Changing the Solvent Affect Sunscreen Performance?

Microsolvation studies of sunscreen chromophores have been conducted to determine how individual solvent molecules can affect the excited state dynamics, and hence the effectiveness of the chromophores as sunscreen filters. Buma and co-workers investigated the effect of complexation of one water molecule on the UVB photodynamics of the cinnamate sunscreen, 2-ethylhexyl-(2*E*)-3-(4-methoxyphenyl)prop-2-enoate (EHMC) by probing the behaviour of a modified form of EHMC, methyl-4-methoxycinnamate (MMC) (Tan et al., 2014). In these experiments, they used resonance enhanced multiphoton ionization spectroscopy of an MMCH_2_O complex formed in a supersonic molecular beam to probe the photodynamics. For bare MMC, they found that absorption of UVB light was not immediately followed by rapid non-radiative decay, as would ideally be the case since photoexcitation resulted in internal conversion to an nπ* state which impeded fast dissipation of the damaging UV light. However, upon H_2_O complexation, the ordering of the key nπ* and ππ* states were reversed so that the bottleneck to ultrafast decay was removed. This led to the conclusion that the microenvironment was intrinsically promoting efficient ultrafast decay for this sunscreen. More recently, Buma and co-workers have used resonance enhanced multiphoton ionization to investigate an isolated complex of the UV filter methyl sinapate with a single water molecule (Fan et al., 2021), again observing how solvent complexation perturbs the electronic properties.

One of the considerable advantages of such gas-phase studies is that they allow direct comparison with high-level computational chemistry calculations. A number of computational studies were performed on cinnamates after the initial MMC study described above, supporting the conclusion that the individual water molecule led to energetic reversal of the crucial nπ* and ππ* states (Xie et al., 2016; Kinoshita et al., 2019).

A detailed insight into how individual water molecules affect sunscreen structure has been obtained using microwave spectroscopy (Domingos and Schnell, 2018). They assigned the global minimum geometry of oxybenzone and identified the two primary water-binding sites formed at either the enol or keto group, measuring their relative stability and internal dynamics. Intriguingly, the water-docking site influences the relative energies of the keto-enol structures that are directly involved in ultrafast energy dissipation. The results show that local structural changes in sunscreen molecules can lead to selective changes in electronic strcture.

Over the last decade, ultrafast transient absorption spectroscopy has been widely applied to probe the fundamental photodynamics of organic sunscreens in solutions. This work complements the gas-phase work described above since it provides insight into how bulk solvation impacts on the intrinsic sunscreen photophysics. A study of diethylamino hydroxybenzoyl in four different solvents (i.e., methanol, dimethyl sulfoxide, acetonitrile, and cyclohexane) by Orr-Ewing and co-workers provides a recent example (Kao et al., 2021). Work in this area has been previously reviewed by Stavros and co-workers (Rodrigues and Stavros, 2018; Holt et al., 2020) so we refer the reader to the reviews for further information.

## Synergistic Effects Through Aggregation of Organic Sunscreens

A recent observation of increased performance of organic sunscreens in the presence of lignin molecules provides further insight into the importance of the local, molecular-level environment on sunscreen efficiency. In a series of experiments, Qian et al. (2016) investigated how the performance of the sunscreens avobenzone and octinoxate changed when they were mixed with organosolv lignin (a mixture of small lignin segments). They observed that the absorption of the mixed solutions increased dramatically, to the extent that the absorption of the mixed solutions was much greater than the sum of the individual component’s absorption. In addition, there were concomitant shifts in the absorption profiles into the UVA region, which is highly notable given how challenging it has proven to identify effective UVA sunscreens. Qian et al. attributed these effects to J-aggregation (a form of π-π* stacking where the angle between the centre of the chromophores is less than 54.7°) between the lignin and the chemical sunscreen molecule (Deng et al., 2011). J-aggregation is known to result in an excitation energy decrease for a π-π* transition, thus producing a substantial redshift in the UV spectrum of the organic sunscreens. It would be important to further characterise the fundamental photophysics associated with these results through gas-phase measurements of complexes of lignin molecular units such as *p*-coumaryl, coniferyl or sinapyl alcohols with organic sunscreen molecules such as avobenzone or octinoxate. Measurements would be possible using either laser-interfaced mass spectrometry or resonance enhanced multiphoton ionization techniques, and could be conducted alongside infrared spectroscopy measurements to verify whether an intermolecular geometry associated with a J-aggregate geometry is present.

In the context of this discussion, recent work from Kohler and co-workers on the photophysics of eumelanin is notable (Grieco et al., 2020). Eumelanin is a biological pigment with sunscreen function and has represented a photophysical puzzle for decades due to uncertainty over the mechanism by which it delivers sunscreen action. Kohler and co-workers have been able to demonstrate that aggregation of chromophore units with diverse oxidation states is key to eumelanin’s ability to dissipate UV radiation.

## Conclusion

Experiments that probe the function of organic sunscreen molecules at the detailed, molecular level have already delivered a much-improved understanding of their safety and photostability. What is particularly striking about these experiments is the extent to which the local chemical environment that the sunscreen molecule interacts with has the ability to significantly perturb sunscreen action. This knowledge has the potential to drive the rational development of new, effective, safe sunscreens and their formulations (Karsili et al., 2014), thus paving the way for a reduction in future melanoma cases. A recent study from Bardeen and co-workers is relevant in this context. They found that encapsulation of avobenzone into sodium dodecylsulfate micelles considerably enhances its photostability (Hanson et al., 2020). This was attributed to the micelle creating an enhanced polar microenvironment, which reduces the propensity of avobenzone to diketonize and hence photodegrade. Further work on the encapsulation of organic sunscreens into rationally designed gels or nanoparticles may well provide a pathway further between to and improved performance (Qiu et al., 2018; Song et al., 2021).

## References

[B1] Acker S. Hloucha M. Osterwalder U. (2014). The Easy Way to Make a Sunscreen. SOFW-Journal 7, 24–30.

[B2] Baker L. A. Horbury M. D. Greenough S. E. Coulter P. M. Karsili T. N. V. Roberts G. M. (2015). Probing the Ultrafast Energy Dissipation Mechanism of the Sunscreen Oxybenzone after UVA Irradiation. J. Phys. Chem. Lett. 6, 1363–1368. 10.1021/acs.jpclett.5b00417 26263136

[B3] Baker L. A. Marchetti B. Karsili T. N. V. Stavros V. G. Ashfold M. N. R. (2017). Photoprotection: Extending Lessons Learned from Studying Natural Sunscreens to the Design of Artificial Sunscreen Constituents. Chem. Soc. Rev. 46, 3770–3791. 10.1039/C7CS00102A 28580469

[B4] Berenbeim J. A. Wong N. G. K. Cockett M. C. R. Berden G. Oomens J. Rijs A. M. (2020a). Sodium Cationization Can Disrupt the Intramolecular Hydrogen Bond that Mediates the Sunscreen Activity of Oxybenzone. Phys. Chem. Chem. Phys. 22, 19522–19531. 10.1039/D0CP03152F 32840272

[B5] Berenbeim J. A. Wong N. G. K. Cockett M. C. R. Berden G. Oomens J. Rijs A. M. (2020b). Unravelling the Keto-Enol Tautomer Dependent Photochemistry and Degradation Pathways of the Protonated UVA Filter Avobenzone. J. Phys. Chem. A. 124, 2919–2930. 10.1021/acs.jpca.0c01295 32208697 PMC7168606

[B6] Bull J. N. Anstöter C. S. Verlet J. R. R. (2020). Fingerprinting the Excited-State Dynamics in Methyl Ester and Methyl Ether Anions of Deprotonated Para-Coumaric Acid. J. Phys. Chem. A. 124, 2140–2151. 10.1021/acs.jpca.9b11993 32105474

[B7] Bull J. N. Silva G. d. Scholz M. S. Carrascosa E. Bieske E. J. (2019). Photoinitiated Intramolecular Proton Transfer in Deprotonated Para-Coumaric Acid. J. Phys. Chem. A. 123, 4419–4430. 10.1021/acs.jpca.9b02023 30964682

[B8] Chang X.-P. Li C.-X. Xie B.-B. Cui G. (2015). Photoprotection Mechanism of P-Methoxy Methylcinnamate: A CASPT2 Study. J. Phys. Chem. A. 119, 11488–11497. 10.1021/acs.jpca.5b08434 26513466

[B9] De Laurentiis E. Minella M. Sarakha M. Marrese A. Minero C. Mailhot G. (2013). Photochemical Processes Involving the UV Absorber Benzophenone-4 (2-Hydroxy-4-Methoxybenzophenone-5-Sulphonic Acid) in Aqueous Solution: Reaction Pathways and Implications for Surface Waters. Water Res. 47, 5943–5953. 10.1016/j.watres.2013.07.017 23953089

[B10] Dean J. C. Kusaka R. Walsh P. S. Allais F. Zwier T. S. (2014). Plant Sunscreens in the UV-B: Ultraviolet Spectroscopy of Jet-Cooled Sinapoyl Malate, Sinapic Acid, and Sinapate Ester Derivatives. J. Am. Chem. Soc. 136, 14780–14795. 10.1021/ja5059026 25295994

[B11] Deng Y. Feng X. Zhou M. Qian Y. Yu H. Qiu X. (2011). Investigation of Aggregation and Assembly of Alkali Lignin Using Iodine as a Probe. Biomacromolecules 12, 1116–1125. 10.1021/bm101449b 21366267

[B12] Domingos S. R. Schnell M. (2018). Wet Sunscreens in the Gas Phase: Structures of Isolated and Microsolvated Oxybenzone. J. Phys. Chem. Lett. 9, 4963–4968. 10.1021/acs.jpclett.8b02029 30091927

[B13] Fan J. Roeterdink W. Buma W. J. (2021). Excited-state Dynamics of Isolated and (Micro)solvated Methyl Sinapate: the Bright and Shady Sides of a Natural Sunscreen. Mol. Phys. 119, e1825850. 10.1080/00268976.2020.1825850

[B14] Fang Y.-G. Li C.-X. Chang X.-P. Cui G. García-Iriepa C. Campos P. J. (2018). Photophysics of a UV-B Filter 4-Methylbenzylidene Camphor: Intersystem Crossing Plays an Important Role. ChemPhysChem 19, 744–752. 10.1002/cphc.201701230 29288547

[B15] Forestier S. (2008). Rationale for Sunscreen Development. J. Am. Acad. Dermatol. 58, S133–S138. 10.1016/j.jaad.2007.05.047 18410799

[B16] Grieco C. Kohl F. R. Hanes A. T. Kohler B. (2020). Probing the Heterogeneous Structure of Eumelanin Using Ultrafast Vibrational Fingerprinting. Nat. Commun. 11, 4569. 10.1038/s41467-020-18393-w 32917892 PMC7486937

[B17] Hanson K. M. Cutuli M. Rivas T. Antuna M. Saoub J. Tierce N. T. (2020). Effects of Solvent and Micellar Encapsulation on the Photostability of Avobenzone. Photochem. Photobiol. Sci. 19, 390–398. 10.1039/C9PP00483A 32100782

[B18] Henley A. Patel A. M. Parkes M. A. Anderson J. C. Fielding H. H. (2018). Role of Photoisomerization on the Photodetachment of the Photoactive Yellow Protein Chromophore. J. Phys. Chem. A. 122, 8222–8228. 10.1021/acs.jpca.8b07770 30234981

[B19] Holt E. L. Krokidi K. M. Turner M. A. P. Mishra P. Zwier T. S. Rodrigues N. d. N. (2020). Insights into the Photoprotection Mechanism of the UV Filter Homosalate. Phys. Chem. Chem. Phys. 22, 15509–15519. 10.1039/D0CP02610G 32602867

[B20] Ignasiak M. T. Houée-Levin C. Kciuk G. Marciniak B. Pedzinski T. Houée-Levin C. (2015). A Reevaluation of the Photolytic Properties of 2-Hydroxybenzophenone-Based UV Sunscreens: Are Chemical Sunscreens Inoffensive? ChemPhysChem 16, 628–633. 10.1002/cphc.201402703 25581220

[B21] Kao M.-H. Venkatraman R. K. Sneha M. Wilton M. Orr-Ewing A. J. (2021). Influence of the Solvent Environment on the Ultrafast Relaxation Pathways of a Sunscreen Molecule Diethylamino Hydroxybenzoyl Hexyl Benzoate. J. Phys. Chem. A. 125, 636–645. 10.1021/acs.jpca.0c10313 33416312

[B22] Karsili T. N. V. Marchetti B. Ashfold M. N. R. Domcke W. (2014). Ab Initio Study of Potential Ultrafast Internal Conversion Routes in Oxybenzone, Caffeic Acid, and Ferulic Acid: Implications for Sunscreens. J. Phys. Chem. A. 118, 11999–12010. 10.1021/jp507282d 25137024

[B23] Kinoshita S.-n. Harabuchi Y. Inokuchi Y. Maeda S. Ehara M. Yamazaki K. (2021). Substitution Effect on the Nonradiative Decay and Trans → Cis Photoisomerization Route: a Guideline to Develop Efficient Cinnamate-Based Sunscreens. Phys. Chem. Chem. Phys. 23, 834–845. 10.1039/D0CP04402D 33284297

[B24] Kinoshita S.-n. Inokuchi Y. Onitsuka Y. Kohguchi H. Akai N. Shiraogawa T. (2019). The Direct Observation of the Doorway 1nπ* State of Methylcinnamate and Hydrogen-Bonding Effects on the Photochemistry of Cinnamate-Based Sunscreens. Phys. Chem. Chem. Phys. 21, 19755–19763. 10.1039/C9CP02914A 31259349

[B25] Kulthanan K. Nuchkull P. Varothai S. (2013). The pH of Water from Various Sources: an Overview for Recommendation for Patients with Atopic Dermatitis. Asia Pac. Allergy 3, 155. 10.5415/apallergy.2013.3.3.155 23956962 PMC3736366

[B26] Li Y. Qiao X. Zhou C. Zhang Y.-n. Fu Z. Chen J. (2016). Photochemical Transformation of Sunscreen Agent Benzophenone-3 and its Metabolite in Surface Freshwater and Seawater. Chemosphere 153, 494–499. 10.1016/j.chemosphere.2016.03.080 27035387

[B27] Losantos R. Funes-Ardoiz I. Aguilera J. Herrera-Ceballos E. García-Iriepa C. Campos P. J. (2017). Rational Design and Synthesis of Efficient Sunscreens to Boost the Solar Protection Factor. Angew. Chem. Int. Ed. 56, 2632–2635. 10.1002/anie.201611627 28128519

[B28] Marlton S. J. P. McKinnon B. I. Hill N. S. Coote M. L. Trevitt A. J. (2021). Electrostatically Tuning the Photodissociation of the Irgacure 2959 Photoinitiator in the Gas Phase by Cation Binding. J. Am. Chem. Soc. 143, 2331–2339. 10.1021/jacs.0c11978 33427467

[B29] Matthews E. Cercola R. Dessent C. (2018). Protomer-Dependent Electronic Spectroscopy and Photochemistry of the Model Flavin Chromophore Alloxazine. Molecules 23, 2036. 10.3390/molecules23082036 PMC6222404 30110962

[B30] Matthews E. Dessent C. E. H. (2017). Experiment and Theory Confirm that UV Laser Photodissociation Spectroscopy Can Distinguish Protomers Formed via Electrospray. Phys. Chem. Chem. Phys. 19, 17434–17440. 10.1039/c7cp02817b 28650510

[B31] Matthews E. Dessent C. E. H. (2018). Observation of Near-Threshold Resonances in the Flavin Chromophore Anions Alloxazine and Lumichrome. J. Phys. Chem. Lett. 9, 6124–6130. 10.1021/acs.jpclett.8b02529 30277786

[B32] Matthews E. Sen A. Yoshikawa N. Bergström E. Dessent C. E. H. (2016). UV Laser Photoactivation of Hexachloroplatinate Bound to Individual Nucleobases In Vacuo as Molecular Level Probes of a Model Photopharmaceutical. Phys. Chem. Chem. Phys. 18, 15143–15152. 10.1039/c6cp01676f 27198464

[B33] Osterwalder U. Sohn M. Herzog B. (2014). Global State of Sunscreens. Photodermatol. Photoimmunol. Photomed. 30, 62–80. 10.1111/phpp.12112 24734281

[B34] Peperstraete Y. Staniforth M. Baker L. A. Rodrigues N. D. N. Cole-Filipiak N. C. Quan W.-D. (2016). Bottom-up Excited State Dynamics of Two Cinnamate-Based Sunscreen Filter Molecules. Phys. Chem. Chem. Phys. 18, 28140–28149. 10.1039/C6CP05205C 27711542

[B35] Proksch E. (2018). pH in Nature, Humans and Skin. J. Dermatol. 45, 1044–1052. 10.1111/1346-8138.14489 29863755

[B36] Qian Y. Qiu X. Zhu S. (2016). Sunscreen Performance of Lignin from Different Technical Resources and Their General Synergistic Effect with Synthetic Sunscreens. ACS Sustain. Chem. Eng. 4, 4029–4035. 10.1021/acssuschemeng.6b00934

[B37] Qiu X. Li Y. Qian Y. Wang J. Zhu S. (2018). Long-Acting and Safe Sunscreens with Ultrahigh Sun Protection Factor via Natural Lignin Encapsulation and Synergy. ACS Appl. Bio Mater. 1, 1276–1285. 10.1021/acsabm.8b00138 34996231

[B38] Robertson P. A. Bishop H. M. Orr-Ewing A. J. (2021). Tuning the Excited-State Dynamics of Acetophenone Using Metal Ions in Solution. J. Phys. Chem. Lett. 12, 5473–5478. 10.1021/acs.jpclett.1c01466 34085833

[B39] Rodrigues N. D. N. Stavros V. G. (2018). From Fundamental Science to Product: A Bottom-Up Approach to Sunscreen Development. Sci. Prog. 101, 8–31. 10.3184/003685018X15166183479666 29422118 PMC10365176

[B40] Serpone N. Dondi D. Albini A. (2007). Inorganic and Organic UV Filters: Their Role and Efficacy in Sunscreens and Suncare Products. Inorg. Chim. Acta 360, 794–802. 10.1016/j.ica.2005.12.057

[B41] Serpone N. (2021). Sunscreens and Their Usefulness: Have We Made Any Progress in the Last Two Decades? Photochem. Photobiol. Sci. 20, 189–244. 10.1007/s43630-021-00013-1 33721254

[B42] Song J. Chen S. Zhao X. Cheng J. Ma Y. Ren S. (2021). Simple, green, Ultrasound-Assisted Preparation of Novel Core-Shell Microcapsules from Octyl Methoxycinnamate and Oligomeric Proanthocyanidins for UV-Stable Sunscreen. RSC Adv. 11, 6374–6382. 10.1039/D0RA09116B PMC8694810 35423144

[B43] Tan E. M. M. Hilbers M. Buma W. J. (2014). Excited-State Dynamics of Isolated and Microsolvated Cinnamate-Based UV-B Sunscreens. J. Phys. Chem. Lett. 5, 2464–2468. 10.1021/jz501140b 26277816

[B44] Uleanya K. O. Cercola R. Nikolova M. Matthews E. Wong N. G. K. Dessent C. E. H. (2020). Observation of Enhanced Dissociative Photochemistry in the Non-native Nucleobase 2-Thiouracil. Molecules 25, 3157. 10.3390/molecules25143157 PMC7397253 32664261

[B45] Wong N. G. K. Berenbeim J. A. Dessent C. E. H. (2019a). Direct Observation of Photochemical Free Radical Production from the Sunscreen 2‐Phenylbenzimidazole‐5‐Sulfonic Acid via Laser‐Interfaced Mass Spectrometry. ChemPhotoChem 3, 1231–1237. 10.1002/cptc.201900149

[B46] Wong N. G. K. Berenbeim J. A. Hawkridge M. Matthews E. Dessent C. E. H. (2019b). Mapping the Intrinsic Absorption Properties and Photodegradation Pathways of the Protonated and Deprotonated Forms of the Sunscreen Oxybenzone. Phys. Chem. Chem. Phys. 21, 14311–14321. 10.1039/C8CP06794E 30680382

[B47] Wong N. G. K. Rankine C. D. Dessent C. E. H. (2021a). Linking Electronic Relaxation Dynamics and Ionic Photofragmentation Patterns for the Deprotonated UV Filter Benzophenone-4. J. Phys. Chem. Lett. 12, 2831–2836. 10.1021/acs.jpclett.1c00423 33719458 PMC8041369

[B48] Wong N. G. K. Rankine C. D. Dessent C. E. H. (2021b). Measurement of the Population of Electrosprayed Deprotomers of Coumaric Acids Using UV-Vis Laser Photodissociation Spectroscopy. J. Phys. Chem. A. 125, 6703–6714. 10.1021/acs.jpca.1c04880 34342453 PMC8389988

[B49] Xie X.-Y. Li C.-X. Fang Q. Cui G. (2016). Mechanistic Photochemistry of Methyl-4-Hydroxycinnamate Chromophore and its One-Water Complexes: Insights from MS-CASPT2 Study. J. Phys. Chem. A. 120, 6014–6022. 10.1021/acs.jpca.6b05899 27398611

